# Assessment HOMA as a predictor for new onset diabetes mellitus and diabetic complications in non-diabetic adults: a KoGES prospective cohort study

**DOI:** 10.1186/s40842-023-00156-3

**Published:** 2023-11-16

**Authors:** Jibeom Lee, Moon-hyun Kim, Ji-Yong Jang, Chang-Myung Oh

**Affiliations:** 1 Department of Biomedical Science and Engineering, Gwangju Institute of Science and Technology, Gwangju, Republic of Korea; 2 Division of Cardiology, Severance Cardiovascular Hospital, Yonsei University College of Medicine, Seoul, Republic of Korea; 3 Division of Cardiology, National Health Insurance Service Ilsan hospital, Goyang, Republic of Korea

**Keywords:** Insulin resistance, Diabetes mellitus, Chronic kidney disease, HOMA-IR

## Abstract

**Background:**

Homeostasis model assessment for insulin resistance (HOMA-IR) is a biomarker for type 2 diabetes mellitus (T2DM). However, the role of HOMA-IR in the non-diabetic is unclear. This study aimed to determine whether IR measured HOMA-IR value is associated with new onset diabetes as well as vascular disease and can be used as an early predictor for diabetes and vascular diseases in non-diabetic participants.

**Methods:**

From a prospective community-based cohort of 10,030 individuals, 4314 individuals younger than 65 years and without diabetes were enrolled and divided into three groups by baseline HOMA-IR tertiles: low (*n* = 1454), moderate (*n* = 1414), and high (*n* = 1446). The primary outcome was new onset T2DM. Secondary outcomes were chronic kidney disease (CKD) and a composite of coronary artery disease, myocardial infarction, and stroke as macrovascular events.

**Results:**

The mean age was 51 years. The prevalence of hypertension and cholesterol and HbA1c were higher in the high HOMA-IR group. New onset T2DM (5.8%) and CKD (12.2%) incidence in the high HOMA-IR group was higher than that in the others. The prevalence of macrovascular events did not differ among groups. High-HOMA-IR was an independent risk factor for new onset T2DM (odds ratio 1.86 [1.17–2.96]; *p* = 0.01) and CKD (1.49 [1.12–1.98]; *p* = 0.01).

**Conclusions:**

High HOMA-IR was an early predictor of new onset T2DM and CKD, regardless of HbA1c in non-diabetic individuals. Further research on the specific cut off value will be needed.

**Supplementary Information:**

The online version contains supplementary material available at 10.1186/s40842-023-00156-3.

## Background

Diabetes mellitus (DM) and its complications are increasing, and the related medical cost is becoming a socio-economic burden worldwide [1]. DM is also closely related to other metabolic diseases, such as dyslipidaemia and fatty liver [2]. Current treatments target individual metabolic diseases, such as hypertension, dyslipidaemia, and diabetes, and are focused on maintenance therapy that prevents disease deterioration rather than preventing and managing the root of the disease. Therefore, besides the high medical costs, the prevalence of metabolic diseases and the associated mortality rates are increasing. To prevent diabetes and its complications, it is important to identify high-risk populations and prevent the disease’s onset in the early and reversible phases.

Insulin resistance (IR), a state of impaired biological response to normal circulating levels of insulin, represents an early pathophysiology of diabetes progression and is associated with micro and macrovascular diseases, as reported by cross-sectional epidemiologic studies [3–6]. Recent short-term observational studies have also suggested that IR may be a risk factor for the development of DM [7]. However, it is not yet clear whether IR is a consequence of diabetes and diabetic complication or a factor leading to it. In this study, we investigated whether IR measured using the homeostatic model assessment for insulin resistance (HOMA-IR) value is associated with new onset diabetes as well as vascular disease and can be used as an early predictor for diabetes and vascular diseases in non-diabetic participants from a large prospective community-based cohort.

## Methods

### Study populations

Data were collected from the Ansan (urban) and Ansung (rural) prospective community-based cohort studies. These studies are part of the Korean Health and Genome Study (KoGES), which is conducted by the Korea Centers for Disease Control and Prevention (Republic of Korea) as a government-funded epidemiological survey to investigate trends in chronic non-communicable diseases and their associated risk factors. From June 2001 to January 2003, adults aged 40–69 years residing in Ansan and Ansung were enrolled. The cohort included a total of 10,030 adults (5018 from Ansung and 5020 from Ansan) who underwent health examination at the Korea University Ansan Hospital and Ajou University Medical Center [8]. The distributions of age and gender were similar to those in the general population. Surveys were conducted every two years through clinical examinations and questionnaires, and a total of six follow-ups were conducted until 2014.

Our study only included participants from the cohort who were under the age of 65 and did not have diabetes. The following were exclusion criteria: age over 65 years at first visit, HbA1c test performed only once at first visit, unavailable HOMA-IR value or diagnosis of diabetes at baseline as follows: 1) HbA1c ≥ 6.5% [9] and 2) taking DM medication at the time of the first visit.

### Covariates

All covariates were based on the time of the first visit and included clinical and biochemical data. Clinical data, such as age, gender, smoking status, hypertension, dyslipidaemia, previous myocardial infarction (MI), previous heart failure, and previous chronic kidney disease (CKD), were obtained using standardised questionnaires by a trained interviewer. Biochemical data, including HbA1c, fasting blood glucose and insulin, lipid profile, and biomarkers reflecting systemic inflammatory status (high sensitivity C-reactive protein), were also obtained, as previously described [10]. Blood samples were obtained after an overnight fast of at least 8 h, and HbA1c levels were measured using high-performance liquid chromatography (Variant II; BioRad Laboratories, Hercules, CA, USA). The glomerular filtration rate (GFR) was calculated using the Modification of Diet in Renal Disease equation at each visit [11]. Body mass index (BMI) was calculated as weight divided by height squared (kg/m^2^).

The fasting insulin and glucose values were used to calculate the values for HOMA-IR, homeostasis model of assessment–β-cell (HOMA–β-cell), and quantitative insulin sensitivity check index (QUICKI) [12, 13]. The subjects were divided into three groups by HOMA-IR value tertiles; the first tertile was 1.37, and the second tertile was 1.84.

### Outcome definition

The primary outcome was new onset DM. New onset DM was diagnosed based on A1C criteria (HbA1C ≥ 6.5%) or taking DM medication during follow up. Secondary outcomes were defined as CKD and macrovascular events. CKD was defined as a creatinine clearance rate of < 60 mL/min/1.73 m^2^. Subjects with CKD at baseline were excluded from the survival analysis. A macrovascular event was defined as a composite of coronary artery disease, MI, and ischemic stroke reported through a questionnaire [3].

### Statistical analysis

All statistical analyses were performed using the SPSS (version 24.0; IBM Corp., Armonk, NY, USA) and R (version 3.1.10; the R Foundation for Statistical Computing, Vienna, Austria) software. Categorical variables are presented as frequencies with percentages and were compared between groups using the chi-square test or Fisher’s exact test. Continuous variables are presented as either mean (± standard deviation) and were compared between groups using one-way analysis of variance.

The cumulative incidences of primary outcomes were compared between groups using the Kaplan–Meier method, with the log rank test. The odds ratios (ORs) and confidence intervals (CIs) for primary and secondary outcomes according to HOMA-IR groups were estimated using multivariable logistic regression analysis after adjustment of variables. Model 1 adjusted clinical factors, such as age, sex, current smoking, hypertension, dyslipidaemia, MI, heart failure, CKD, and HbA1c. Model 2 additionally adjusted laboratory variables, such as high-density lipoprotein (HDL)-cholesterol, low-density lipoprotein (LDL)-cholesterol, C-reactive protein, HOMA-β-cell, GFR, and BMI. Statistical significance was considered at *p*-value < 0.05.

### Ethical considerations

The Institutional Review Board of Gwangju Institute of Science and Technology (South Korea) approved the study protocol (IRB No. 20200414-EX-01-02). All research procedures were performed in accordance with the relevant guidelines and regulations. All participants volunteered for the Ansan and Ansung studies and provided written informed consent.

## Results

### Baseline characteristics

**Fig. 1:**
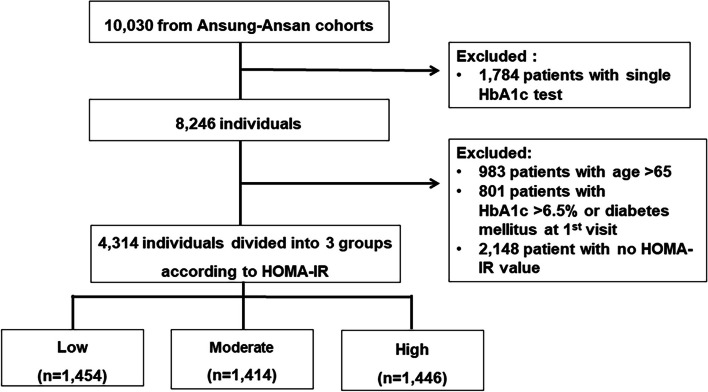
Flow chart of study design

Among the 10,030 individuals in the study cohort, 1784 individuals who only tested for HbA1c once were excluded. In addition, participants were excluded if they were > 65 years old at baseline (*n* = 983) or diagnosed with DM at baseline (*n* = 801) and without an HOMA-IR value (*n* = 2148). Finally, 4314 individuals were enrolled in this study (Fig. 1). The participants were divided into low (*n* = 1454), moderate (*n* = 1414), and high (*n* = 1446) HOMA-IR groups based on HOMA-IR value tertiles.

The subjects were followed for a median interval of 9.9 years (interquartile range: 9.1–10.0 years). Clinical and biochemical baseline characteristics of the HOMA-IR groups are presented in Table 1. The mean age was 51 years in all HOMA-IR groups, and the proportions of men in the low, moderate, and high HOMA-IR groups were 56.6, 47.3, and 45.4%, respectively. The prevalence of hypertension was higher in the high HOMA-IR group (16.7%) than in the moderate (10.7%) and low (8.8%) HOMA-IR groups, but the prevalence of dyslipidaemia, previous MI, previous heart failure, and CKD were not significantly different among the HOMA-IR groups.

**Table 1:** Clinical and laboratory characteristics of the study cohort

HOMA-IR group						
	Low (*n* = 1454)	Moderate (*n* = 1414)	High (*n* = 1446)	*p*	*p’*	*p”*
Age (years)	51.5 ± 7.8	50.7 ± 7.6	50.6 ± 7.6	0.001	0.001	1.00
Male	823 (56.6)	669 (47.3)	657 (45.4)	< 0.001	< 0.001	0.33
Current smoking	459 (32.1)	331(23.8)	312(21.8)	< 0.001	< 0.001	0.23
Hypertension	128 (8.8)	151 (10.7)	242 (16.7)	< 0.001	< 0.001	< 0.001
Dyslipidaemia	23 (1.6)	25 (1.8)	36 (2.5)	0.18	0.09	0.19
Previous MI	6 (0.4)	14 (1.0)	10 (0.7)	0.18	0.33	0.42
Previous heart failure	3 (0.2)	2 (0.1)	2 (0.1)	0.88	0.99	0.99
Previous CKD	28 (1.9)	39 (2.8)	43 (3.0)	0.17	0.07	0.74
Laboratory variables						
Total cholesterol (mg/dL)	193 ± 34	196 ± 34	203 ± 36	< 0.001	< 0.001	< 0.001
Triglyceride (mg/dL)	128 ± 84	139 ± 93	168 ± 116	< 0.001	< 0.001	< 0.001
HDL-cholesterol (mg/dL)	52 ± 12	50 ± 12	47 ± 11	< 0.001	< 0.001	< 0.001
LDL-cholesterol (mg/dL)	116 ± 33	118 ± 32	122 ± 34	< 0.001	< 0.001	0.14
HbA1c (%)	5.5 ± 0.3	5.5 ± 0.3	5.6 ± 0.4	< 0.001	< 0.001	< 0.001
HbA1c (mmol/mol)	37 ± 2	37 ± 2	38 ± 2	< 0.001	< 0.001	< 0.001
CRP (mg/L)	0.22 ± 0.55	0.23 ± 0.85	0.22 ± 0.26	0.89	1.00	1.00
Fasting glucose (mg/dL)	89 ± 7	91 ± 7	94 ± 8	< 0.001	< 0.001	< 0.001
Fasting insulin (U/mL)	6.2 ± 1.4	7.5 ± 1.5	10.1 ± 3.1	< 0.001	< 0.001	< 0.001
HOMA-IR^c^	1.02 ± 0.42	1.51 ± 0.45	2.43 ± 1.45	< 0.001	< 0.001	< 0.001
HOMA-β-cell	90 ± .32	102 ± 33	122 ± 47	< 0.001	< 0.001	< 0.001
QUICKI	0.37 ± 0.01	0.35 ± 0.01	0.34 ± 0.01	< 0.001	< 0.001	< 0.001
GFR (mL/min/1.73 m^2^)	96 ± 20	95 ± 21	95 ± 21	0.26	0.43	1.00
BMI (kg/m^2^)	23.2 ± 2.8	24.3 ± 2.8	25.8 ± 2.9	< 0.001	< 0.001	< 0.001

Values are presented as number (%) or mean ± SD. *p*’ is for the comparison between the low and high HOMA-IR groups. *p*” is for the comparison between the moderate and high HOMA-IR groups

*Abbreviations:* *BMI *Body mass index, *CAD* Coronary artery disease, *CKD *Chronic kidney disease, *CRP *C-reactive protein, *GFR *Glomerular filtration rate, *HbA1c *Glycated haemoglobin, *HDL *high-density lipoprotein, *HOMA-IR *Homeostasis model assessment for insulin resistance, *MI *Myocardial infarction, *LDL *Low-density lipoprotein, *QUICKI *Quantitative insulin sensitivity check index

The mean values of HOMA-IR were 1.02, 1.51, and 2.43 and those of HOMA-β-cell were 90, 102, and 122, respectively, in the low, moderate, and high groups. The high HOMA-IR group had a higher mean HbA1c, total cholesterol, triglyceride, and LDL-cholesterol and lower HDL-cholesterol than the other groups, although all values were within normal ranges. In contrast, C-reactive protein and baseline GFR values were not different among the three groups.

### Primary outcomes for the HOMA-IR groups

**Fig. 2:**
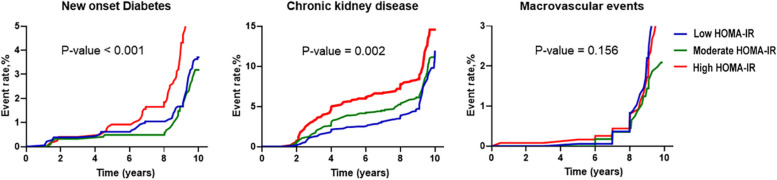
Kaplan–Meier survival curves according to HOMA-IR groups

The primary outcome was defined as new onset DM, and the secondary outcomes were defined as CKD and macrovascular events. New onset DM was observed in a total of 164 participants, and more participants were newly diagnosed with diabetes in the high HOMA-IR group (*n* = 84, 5.8%) than in the other groups (*p*-value < 0.001). CKD was observed in a total of 425 participants, and more participants were diagnosed in the high HOMA-IR group (*n* = 173, 12.2%) than in the other groups (*p*-value = 0.002). On the contrary, macrovascular events, including coronary artery disease, MI, and ischemic stroke, were observed in a total of 102 participants, and there was no significant difference in the incidence of macrovascular events among the HOMA-IR groups (Table 2, Fig. 2).

**Table 2:** Primary and secondary outcomes according to HOMA-IR groups

HOMA-IR groups						
	Low (*n* = 1454)	Moderate (*n* = 1414)	High (*n* = 1446)	*p*-value	p’	p”
New onset Diabetes	43 (3.0)	37 (2.6)	84 (5.8)	< 0.001	< 0.001	< 0.001
Chronic kidney disease	123 (8.5)	129 (9.3)	173 (12.2)	0.002	0.001	0.007
Macrovascular events	41 (2.9)	26 (1.8)	35 (2.4)	0.156	0.407	0.263
Coronary artery disease or MI	29 (2.0)	15 (1.5)	20 (1.4)	0.052	0.095	0.610
Ischemic stroke	12 (0.8)	11 (0.8)	15 (1.0)	0.809	0.707	0.511

Values are presented as number (%), *p*-values were calculated using Kaplan–Meier survival analysis with the log rank test. P′ is for the comparison between the low and high HOMA-IR groups. p” is for the comparison between the moderate and high HOMA-IR groups

### HOMA-IR as an independent predictor of new onset diabetes and CKD

Table 3 shows the OR for primary and secondary outcomes in the HOMA-IR groups. After adjusting for clinical risk factors in model 1, high HOMA-IR was found to be a marginal risk factor for new onset DM (OR: 1.42, 95% CI: 0.95–2.14, *p*-value = 0.09). Additionally, after adjusting for baseline laboratory variables and BMI in model 2, high HOMA-IR was a significant risk factor for the development of DM (OR: 1.86, 95% CI: 1.17–2.96, *p*-value = 0.01). The baseline HbA1c level was also a significant risk factor for new onset diabetes. The results for CKD were similar to those for new onset diabetes. In model 1, the OR for newly diagnosed CKD in the high HOMA-IR group was 1.42 (95% CI: 1.10–1.84, *p*-value = 0.01) and in model 2, the OR for newly diagnosed CKD in the high HOMA-IR group was 1.49 (95% CI: 1.12–1.98, *p*-value = 0.01). In contrast, the HbA1c level was not a risk factor for the development of CKD. There was no significant difference in ORs between high HOMA-IR and HbA1c values for macrovascular events in both models. BMI, as an indicator of metabolic disease, is also a significant risk factor for new onset DM but not vascular events (Table S1 of Additional file 1).

**Table 3:** Multivariate analysis of primary and secondary outcomes in the HOMA-IR groups

	Model 1		Model 2	
	Odds ratio (95% CI)	*p*-value	Odds ratio (95% CI)	*p*-value
New onset DM				
HbA1c (%)	28.1(16.9–46.5)	< 0.001	28.9 (16.9–49.8)	< 0.001
Low HOMA-IR	Reference		Reference	
Moderate HOMA-IR	0.78 (0.49–1.26)	0.31	0.91 (0.55–1.51)	0.72
High HOMA-IR	1.42 (0.95–2.14)	0.09	1.86 (1.17–2.96)	0.01
Chronic kidney disease				
HbA1c (%)	1.15 (0.85–1.55)	0.36	1.10 (0.80–1.52)	0.56
Moderate HOMA-IR	1.07(0.81–1.40)	0.65	1.01 (0.75–1.36)	0.96
High HOMA-IR	1.42 (1.10–1.84)	0.01	1.49 (1.12–1.98)	0.01
Macrovascular event				
HbA1c (%)	1.43 (0.81–2.54)	0.22	1.31 (0.71–2.44)	0.39
Moderate HOMA-IR	0.67 (0.41–1.11)	0.12	0.64 (0.38–1.09)	0.11
High HOMA-IR	0.84 (0.52–1.34)	0.46	0.91 (0.53–1.56)	0.74

Model 1, adjusted for HbA1c, age, sex, current smoking, hypertension, dyslipidaemia, myocardial infarction, heart failure, chronic kidney disease; Model 2, adjusted for factors in model 1 as well as high-density lipoprotein cholesterol, low-density lipoprotein cholesterol, C-reactive protein, homeostasis model assessment of β-cell function, glomerular filtration rate, and body mass index

Changes in HOMA-IR and HOMA-β-cell values between the baseline and last visit were calculated (Fig. S1 of Additional file 1). The HOMA-IR value was significantly increased in the low and moderate HOMA-IR groups compared to that in the high HOMA-IR group. HOMA-β-cell decreased over time, regardless of the group. The decrease in HOMA-β-cell was significantly higher in the high HOMA-IR group than in the low and moderate HOMA-IR groups.

## Discussion

Analysis of a non-diabetic middle-aged population from a large prospective, community-based cohort with a long term follow up revealed a significantly higher prevalence of new onset diabetes and CKD in the high HOMA-IR group than in the other groups. High baseline HOMA-IR was an independent risk factor for both new onset diabetes and CKD regardless of the HbA1c level. However, there was no association between high HOMA-IR and macrovascular events.

Many previous studies have shown the relationship between IR and diabetes [3, 4, 6]. IR refers to reduced responsiveness to insulin in tissues that take up glucose, such as liver, skeletal muscle, and adipose tissue [14, 15]. In compensation for IR, the synthesis of insulin in β cells increases and hyperinsulinemia occurs, leading to impaired glucose disposal [15]. Type 2 diabetes mellitus (T2DM) is induced when there is a combination of insufficiencies in β cell mass and function to meet the demands of IR. A high serum glucose level inhibits the proliferation and de-differentiation of β cells through a process called “glucotoxicity,” which gradually leads to reduced insulin secretion [16]. In the high HOMA-IR group, the HOMA-β-cell value for β cell function decreased more than that in the other groups in our study. Given that individuals with T2DM have a β cell mass and function occasionally preserved within normal range in the early period of T2DM progression, β cell mass and function insufficiencies were relative rather than absolute. Although an insufficient β cell mass is essential for the development of T2DM [17], it is difficult to accurately measure β cell mass in living people, and insulin secretion capacities widely vary; therefore, β cell mass has limited use as a biomarker for new onset diabetes.

In contrast, IR is commonly observed in most T2DM patients and in individuals with impaired glucose tolerance. IR is reportedly the strongest predictor of T2DM, and diabetes can be prevented by improving IR [5]. IR begins from the very early stage of diabetes and can thus be used as an early biomarker to estimate the risk of new onset diabetes. A recent Saku study [4] assessed IR and diabetes in 2209 non-diabetic patients. Changes in HOMA-IR were measured in the non-diabetic patients and showed that the incidence of T2DM was high when the changes in HOMA-IR were moderate or high. The Saku study also showed that IR had a strong impact on the development of diabetes. This finding is line with our study. Our study showed a significant relationship between high HOMA-IR and new onset diabetes, even in non-diabetic patients from a larger prospective community-based cohort with ten years of follow up.

BMI is a metabolic disease parameter and is associated with IR and diabetes [18]. In our study, BMI was found to be a significant risk factor for new onset DM but not vascular events. Because BMI is calculated based only on height and weight, it does not seem to represent metabolic status more sensitively than other parameters, such as visceral fat and waist-to-hip ratio [19–21]. Vascular disease is also directly affected by factors other than metabolic disease, such as high blood pressure.

Another important finding of the present study was that high HOMA-IR value was found to be an independent risk factor for CKD among non-diabetic individuals, whereas HbA1c was not. Various studies have been conducted on the relationship between CKD and IR [22–25]. CKD, itself characterised by a low-grade inflammatory state, can cause IR and vice versa. CKD and IR adversely affect each other, accelerating the deterioration of renal function [23]. There are several mechanisms that have been suggested to underly the relationship between CKD and IR, one of which is hyperinsulinemia, which increases oxidative stress, protein glycosylation oxidation, and lipid peroxidation [5]. Hyperinsulinemia causes glomerular hyperfiltration, endothelial dysfunction, and increased vascular permeability. IR with oxidative stress and inflammation is thought to play roles in microalbuminuria development and kidney function impairment [5]. In addition to hyperinsulinemia, inappropriate activation of the renin-angiotensin-aldosterone system may cause renal insufficiency [26]. Eventually, IR can lead to glomerulosclerosis and tubulointerstitial injury. A 3-year prospective cohort study with 7200 patients showed that the incidence of CKD and rate of decrease of eGFR were higher in the high HOMA-IR group with metabolic syndrome [27]. Our study also showed that high HOMA-IR was an independent risk factor for CKD after adjusting for multiple risk factors, including HbA1c and baseline GFR. HbA1c was not a risk factor for CKD in our study. This might be due to the fact that the HbA1c value in our study was within the normal range, unlike the 7% HbA1c standard value for predicting microvascular complications in the UKPDS study [26].

The incidence of macrovascular events did not differ among the HOMA-IR groups. Some studies have reported a relationship between IR and cardiovascular events. However, it is difficult to directly compare these with our study, since most of these previous studies have a cross-sectional design and involved a few participants or participants who already had atherosclerosis identified as a high-risk factor [22, 27–29]. The clinical significance of IR for cardiovascular disease may more likely be as a factor accelerating disease progression in patients with certain risk factors, such as CKD, rather than as an independent risk factor [22, 29]. Another issue with our study was that it focused on a population that was relatively young and healthy, and thus, the risk of cardiovascular events was very low. Major vascular complications begin to develop about 10 years after diabetes diagnosis. Follow-up of the cohort in the present study is still ongoing; thus, we hope to observe very long-term cardiovascular events and influence of high baseline HOMA-IR value.

There are several limitations to this study. First, IR was evaluated using only HOMA-IR. The gold standard for evaluating IR is the hyperinsulinemia-euglycemic glucose clamp technique [12], but it is clinically difficult to implement and even more difficult to apply in large-scale cohort studies. In contrast, HOMA-IR is widely used to measure IR and has yielded reliable results in many studies [28, 29]. Second, clinical data were obtained through standardised questionnaires by a trained interviewer. However, the incidence of macrovascular events in this relatively healthy cohort was lower than that among people with diabetes. Large cohort studies routinely use standardised questionnaires, and the incidence of macrovascular events in our Korean cohort was similar to that in other ethnic groups without diabetes [30]. Third, the absence of data regarding other microvascular events, such as retinopathy, could be a limitation, although the expected incidences of end-stage DM-related microvascular events in our cohort are very low, as the participants did not have diabetes at baseline. It is difficult to infer the effect of specific medication on clinical events because of the lack of medication data. As mentioned above, participants included in this analysis had a very low incidence of risk factors; thus, the effect of drugs in our cohort is expected to be insignificant.

## Conclusions

Our findings indicate that high baseline HOMA-IR has a significant relationship with the development of T2DM and CKD and is an independent risk factor for both new onset T2DM and CKD, regardless of the HbA1c level in a healthy middle-age population. However, we found no association between high HOMA-IR and macrovascular events. This suggests that HOMA-IR measurement can be used as a biomarker to identify people at risk for T2DM development. Further studies are needed to define a HOMA-IR cut off value as a new onset T2DM prediction marker and determine its association with macrovascular disease through long-term follow up.

### Supplementary Information

**Additional file 1: Table S1. **Odds ratios for body mass index in multivariate analysis of primary and secondary outcomes. **Figure S1.** Progressive change of HOMA-IR and HOMA-beta cell function during follow-up (* indicated *p* <0.001).

## Data Availability

The corresponding author, Ji-Young Jang, may provide the data that back up the conclusions of this study upon request. Researchers who wish to use the integrate dataset can access the KoGES epidemiological data online sharing system (https://nih.go.kr/ko/main/contents.do?menuNo=300564). A guidebook for using the integrated dataset is also available in the online sharing system.

## Abbreviations

BMI: Body mass index
CAD: Coronary artery disease
CKD: chronic kidney disease
CRP: C-reactive protein
GFR: Glomerular filtration rate
HbA1c: Glycated haemoglobin
HDL: High-density lipoprotein
HOMA-IR: Homeostasis model assessment for insulin resistance
MI: Myocardial infarction
LDL: Low-density lipoprotein
QUICKI: Quantitative insulin sensitivity check index
T2DM: Type 2 diabetes mellitus
UKPDS: The UK Prospective Diabetes Study
