# Supplementary material for: Assessment HOMA as a predictor for new onset diabetes mellitus and diabetic complications in non-diabetic adults: a KoGES prospective cohort study
Source: Clin Diabetes Endocrinol. 2023 Nov 16;9:7. doi: 10.1186/s40842-023-00156-3 (PMC10652621; doi:10.1186/s40842-023-00156-3)

**Table S1. Odds ratios for body mass index in multivariate analysis of primary and secondary outcomes.**

|  | Odds ratio (95% CI) for Body mass index | p-value |
| --- | --- | --- |
| New onset DM | 1.15 (1.07-1.24) | < 0.001 |
| Chronic kidney disease | 1.02 (0,97-1.06) | 0.54 |
| Macrovascular event | 0.99 (0.99-1.00) | 0.81 |

Model 1, adjusted for HbA1c, age, sex, current smoking. hypertension, dyslipidemia, myocardial infarction, heart failure, chronic kidney disease; Model 2, adjusted for factors in model 1 as well as high density lipoprotein cholesterol, low density lipoprotein cholesterol, C reactive protein, homeostasis model assessment of β-cell function, glomerular filtration rate, and body mass index

**Figure S1. Progressive change of HOMA-IR and HOMA-beta cell function during follow-up (* indicated p <0.001)**


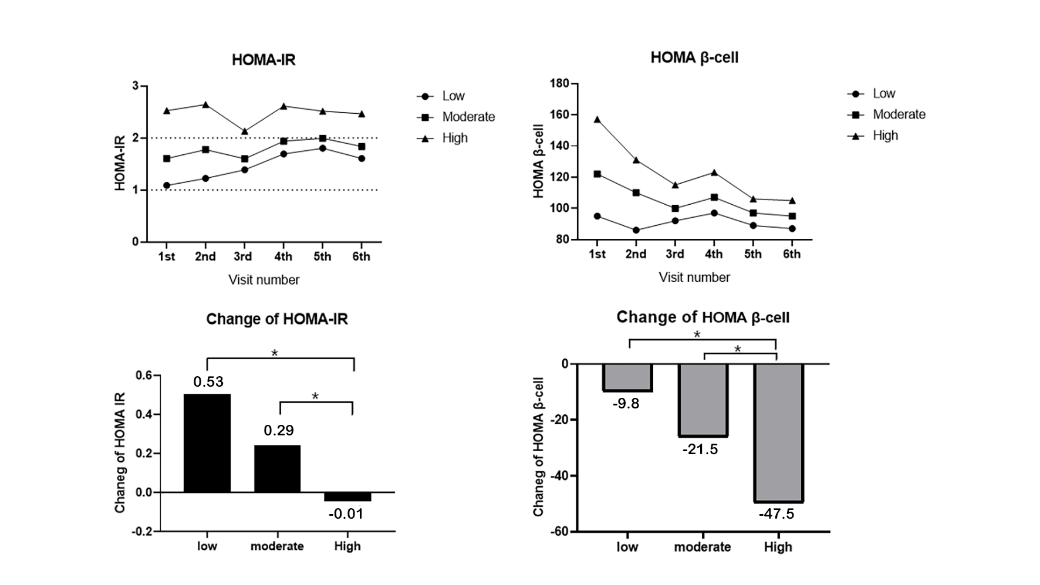

Supplement: Supplementary file 1 — Additional file 1: Table S1. Odds ratios for body mass index in multivariate analysis of primary and secondary outcomes. Figure S1. Progressive change of HOMA-IR and HOMA-beta cell function during follow-up (* indicated p <0.001). [file 40842_2023_156_MOESM1_ESM.docx]
